# Differences in Geographic Patterns of Absolute and Relative Black–White Disparities in Stroke Mortality in the United States

**DOI:** 10.5888/pcd19.220081

**Published:** 2022-10-06

**Authors:** Aspen Flynn, Adam S. Vaughan, Michele Casper

**Affiliations:** 1 Division for Heart Disease and Stroke Prevention, National Center for Chronic Disease Prevention and Health Promotion, Centers for Disease Control and Prevention, Atlanta, Georgia

**Figure:**
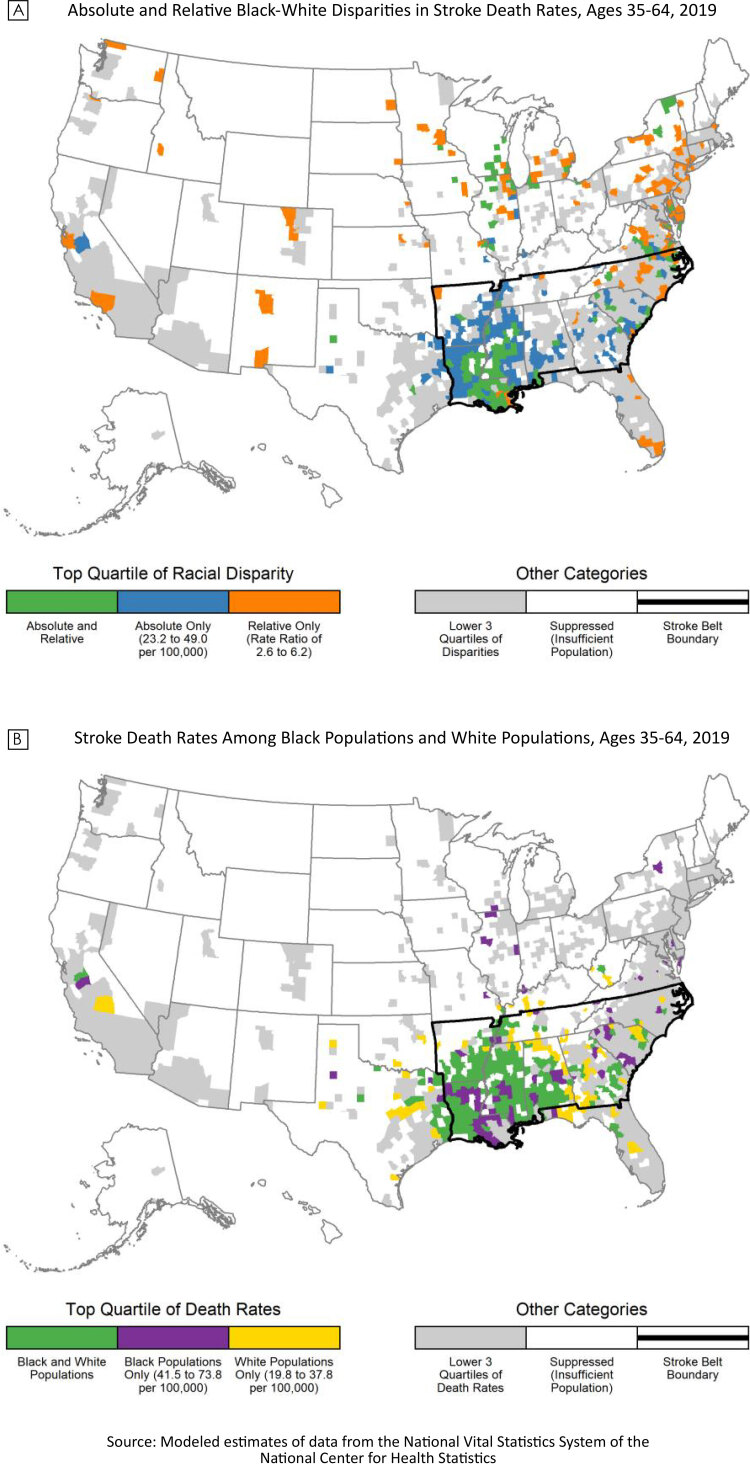
Absolute and relative Black–White disparities in stroke death rates for people aged 35 to 64 years, 2019 (Map A), and stroke death rates for Black populations and White populations for people aged 35 to 64 years, 2019 (Map B). Source: National Center for Health Statistics.

## Background

In the US, racial disparities in stroke death rates are particularly large among working age adults, for whom the stroke death rate in 2019 among non-Hispanic Black adults aged 35 to 64 years was 2.4 times that of their non-Hispanic White counterparts (1,2). These national disparities occur in the context of marked local variation in stroke death rates among both Black and White populations. Within the Stroke Belt (a band of southern US states with high stroke mortality), stroke death rates for both Black and White populations are persistently high (3). However, county-level racial disparities in stroke death rates have not been documented. These data are critical to addressing racial inequities in stroke mortality by shaping public health agendas, engaging communities, and guiding prioritization and development of programs, interventions, and policies (2,4). Therefore, we calculated race-specific stroke death rates in 2019 for adults aged 35 to 64 years and mapped the geographic variation of the largest absolute and relative Black–White disparities in stroke death rates (Map A) and of the highest stroke death rates for Black populations and White populations (Map B).

## Data and Methods

We obtained stroke death counts (International Classification of Diseases, 10th revision codes I60-I69) and total population for people aged 35 to 64 years by county of residence for 2019 from the National Vital Statistics System of the National Center for Health Statistics (5,6). We used a Bayesian conditional autoregressive model to estimate county-level stroke death rates for non-Hispanic Black and White populations aged 35 to 64 years in 2019 (7). This model smooths data across neighboring counties to generate reliable, precise estimates of county-level death rates, even for counties with small populations (7,8). Using these rates, we calculated absolute and relative Black–White stroke mortality disparities for each county. We then mapped the counties in the top quartile for race-specific stroke death rates and Black–White stroke mortality disparities. For a county to be included in this analysis, we required that, for both Black and White populations, the estimated stroke death rate be reliable (ie, the rate’s precision as defined by the width of the 95% credible interval was less than the point estimate) and the population was greater than 1,000 in 2019. These requirements ensured that we only reported reliable heart disease death rates for sufficiently large populations. All rates were age-standardized to the 2010 US population. We used R version 4.1.1 for data analysis and map creation (9).

## Highlights

The largest absolute and relative Black–White disparities in stroke death rates among adults aged 35 to 64 years had different and opposing county-level geographic patterns (Map A). Counties in the top quartile of absolute Black-White disparities (rate difference 23.2 to 49.0 deaths per 100,000 population) were concentrated in the South in the well-established Stroke Belt, where stroke death rates were high for both Black and White populations. Counties in the top quartile of relative disparities (rate ratio 2.6 to 6.2) were scattered across the mid-Atlantic, Northeast, and Great Lakes region. Counties in the top quartile for both absolute and relative disparities were located primarily in the Mississippi Delta region. Counties in the top quartile of stroke death rates for both Black and White populations were concentrated in the South, primarily Louisiana, the Mississippi Delta region, and western Alabama (Map B).

The similarity of geographic patterns for large absolute Black–White disparities and high race-specific stroke death rates (both concentrated in the Stroke Belt) stems from the calculation of absolute disparities. Given the presence of high stroke death rates for both Black populations and White populations in the Stroke Belt (Appendix), the absolute difference in rates must, by definition, be higher in this region (Appendix). In contrast, the largest relative Black–White disparities occurred primarily in counties with lower stroke death rates. A majority of the counties with large relative disparities (64.1%) are located outside the Stroke Belt. Mimicking the geographic pattern of counties with high stroke death rates for both Black populations and White populations, counties in Mississippi and Louisiana are in the top quartile of both absolute and relative disparities.

## Action

The markedly different geographic patterns of absolute and relative Black–White disparities in stroke mortality among adults aged 35 to 64 years demonstrate the importance of examining both measures of disparities, along with race-specific rates, when prioritizing efforts to eliminate racial inequities in stroke mortality. Absolute and relative disparity measures provide different, but complementary, documentation necessary to fully address racial inequities in stroke mortality (10). Large absolute disparities highlight areas with high underlying race-specific stroke death rates, whereas large relative disparities draw attention to areas where race-specific death rates may be lower but inequities are still large.

Racial inequities are most commonly measured as relative disparities (11). For stroke mortality, the observation that counties with the largest relative Black–White disparities tend to have lower race-specific stroke death rates suggests that the conditions contributing to lower rates are not extended equitably across racial groups. Using only relative disparity as the basis for programs and policies focused on eliminating Black–White disparities in stroke mortality, however, would miss many counties in the Stroke Belt where stroke death rates are high for both Black populations and White populations. Conversely, using only absolute disparity as the metric for efforts to eliminate Black–White inequities in stroke mortality would miss many communities outside the Stroke Belt with lower stroke death rates yet substantial excess mortality among Black populations. Finally, programs and policies that focus on areas with large disparities in both relative and absolute terms will reach a smaller, albeit important, subset of counties.

## Appendix County-level race-specific stroke death rates per 100,000 for Black populations and White populations aged 35 to 64 years, 2019

Map A shows county-level stroke death rates for Black populations aged 35 to 64 years, and Map B shows county-level stroke death rates for White populations aged 35 to 64 years. Quartile cutpoints are based on the race-specific distributions of stroke death rates per 100,000 population. Only counties that met the inclusion criteria for the study are included on the maps.
